# On analysis of topological indices and heat of formation for benzyl sulfamoyl network via curve fitting model

**DOI:** 10.1038/s41598-024-66579-9

**Published:** 2024-07-05

**Authors:** Hong Yang, Muhammad Farhan Hanif, Muhammad Kamran Siddiqui, Muhammad Faisal Hanif, Ayesha Maqbool, Mohamed Abubakar Fiidow

**Affiliations:** 1 School of Computer Science, Chengdu University, Chengdu, China; 2 Department of Mathematics and Statistics, The University of Lahore, Lahore Campus, Lahore, Pakistan; 3 Department of Mathematics, COMSATS University Islamabad, Lahore Campus, Lahore, Pakistan; 4 Department of Mathematical Sciences, Faculty of Science, Somali National University, Mogadishu Campus, Mogadishu, Somalia

**Keywords:** Topological indices, Statistical analysis, Zagreb type indices, Forgotten index, Heat of formation, Curve fitting model, Benzyl sulfamoyl network, Applied mathematics, Physical chemistry

## Abstract

The study explores the intricate relationship between topological indices and the heat of formation in the benzyl sulfamoyl network. Topological indices of benzyl sulfamoyl networks are studied and also emphasize their properties statistically. The benzyl sulfamoyl has unique properties due to its crystalline structure and it is used in the form of artificial substance. We analyze the distributions and correlations of the benzyl sulfamoyl network with others by using statistical methods and also build a computational analysis for topological indices. The findings show a strong association between the variables, indicating that topological indices may be used to accurately predict thermodynamic characteristics and improve the effectiveness of molecular modelling and simulation procedures.

## Introduction

A graphical representation made up of points and lines is called a graph. Chemists use graphs to correlate the formula of familiar chemical compounds. In a molecular graphs, atoms are represented by vertices and edges are represented the bonds between atoms which are connected to the respective vertices. The total number of edge connected with a vertex $\vartheta$ is called its degree and is denoted by $\Im (\vartheta )$. In mathematics a graph is defined by an ordered pair of vertices and edges $G=(V, E)$: in this ordered pair *V* is a set of vertices, and *E* is represented by edges^1^. A correspondence of molecular structure’s topologies and their chemical and physical properties their biological traits, involving melting and boiling points, and Medication toxicity is exposed through chemical Compatibility^2^. A unique class of topological indices is vertex-degree-based indices which are determined by the vertex degree in the graph^3^. To understand the relations of molecular structure and their physical and chemical properties chemical engineer use General Randic index^4^.

Topological indices have become indispensable in the field of chemical graph theory for comprehending the structural characteristics of molecular networks. These indices, which measure several facets of molecular topology, are essential for understanding how molecule structure and physicochemical characteristics interact. The benzyl sulfamoyl network is unique among the many molecular networks that have been researched because of its important uses in materials science and pharmaceuticals^5^. Arockiaraj et al.^6^ discuss the topological Indices of hydrogen bonded benzo-trisimidazole. Liu et al.^7^ discuss the topological indices of molecular graphs. Ahmad et al.^8,9^ discuss the theoretical study of energy of phenylene and anthracene. Nadeem et al.^10^ computed the topological aspects of molecular structures. Liu et al.^11^ computed the descriptors and graph entropy of Zeolite ACO. Koam et al.^12^ determined the valency-based topological descriptor for hexagon star networks.

We explore the association between several topological indices and the heat of creation in the benzyl sulfamoyl network in this work using a curve fitting model. In particular, we examine the relationship between Zagreb type indices, the forgotten index, and other pertinent topological descriptors with the heat of creation^13^. Our goal is to create prediction models that can precisely calculate the heat of formation from topological indicators by utilising statistical approaches. Milan Randi$\acute{c}$ discovered its initiation in 1975 as Randi$\acute{c}$ index which is defined as the sum of the convertible of the square root of the product of degrees of vertices which are joined by an edge^14^. To find the computable structure-activity Connections (*CSAC*) and evaluation to find the biological activity of chemical compounds^15^. It gives us the information about arrangement of atoms in a molecule. In 1998 Amic et al.^16^ and Bollob$\acute{{\rm a}}$s et al.^17^, work separately and defined the generalized Randi$\acute{c}$ index as:

1 $$\begin{aligned} R_{\alpha }(G)=\sum \limits _{\vartheta \varpi \in E(BS_b)}\big (\Im (\vartheta )\times \Im (\varpi )\big )^{\alpha },\quad \text {Where } \alpha = 1,\, -1,\, \frac{1}{2},\, -\frac{1}{2} \end{aligned}$$

The above-given index tells us the numerical illustration and connection of atoms inside the molecule. The index is determined by sum-up the products of the bond orders between atom pairs in a molecule^18,19^. This descriptor contributes to the quantification of the molecular structure’s complexity and aids in the prediction of several attributes, including physicochemical traits and biological activity^20^. These properties help us to measure the complexity of the molecular structure and enable us to estimate the different characteristics, such as physical and chemical properties, also biological activity. Estrada et al.^21,22^ defined a new idea of an index which is known as the atom bond connectivity index:

2 $$\begin{aligned} ABC(G)= \sum \limits _{\vartheta \varpi \in E(BS_b)}\sqrt{\frac{\Im (\vartheta )+\Im (\varpi )-2}{\Im (\vartheta )\times \Im (\varpi )}}. \end{aligned}$$

Vukicevic et al.^23^, discover the geometric arithmetic index as follows:

3 $$\begin{aligned} GA(G)= \sum \limits _{\vartheta \varpi \in E(BS_b)}\frac{2\sqrt{\Im (\vartheta )\times \Im (\varpi )}}{\Im (\vartheta )+\Im (\varpi )}. \end{aligned}$$

Gutman^24,25^ has made substantial involvement in the subject of chemical graph theory introducing the first and second Zagreb indices theoretically as:

4 $$\begin{aligned} M_{1}(G)= \sum \limits _{\vartheta \varpi \in E(BS_b)}(\Im (\vartheta )+\Im (\varpi )). \end{aligned}$$

5 $$\begin{aligned} M_{2}(G)= \sum \limits _{\vartheta \varpi \in E(BS_b)}(\Im (\vartheta )\times \Im (\varpi )). \end{aligned}$$

In 2013 Shirdal et al.^26^ defined the hyper Zagreb indicator as:

6 $$\begin{aligned} HM(G)= \sum \limits _{\vartheta \varpi \in E(BS_b)}(\Im (\vartheta )+\Im (\varpi ))^{2}. \end{aligned}$$

Furtula and Gutman^27^ introduced the forgotten topological index, as follows:

7 $$\begin{aligned} F(G)= \sum \limits _{\vartheta \varpi \in E(BS_b)}(\Im (\vartheta )^{2}+\Im (\varpi )^{2}). \end{aligned}$$

Redefined Zagreb type indicator introduced by Rajini^28^ which is a new idea for any graph as:

8 $$\begin{aligned} ReZG_{1}(G)= \sum \limits _{\vartheta \varpi \in E(BS_b)}\frac{\Im (\vartheta )+\Im (\varpi )}{\Im (\vartheta )\times \Im (\varpi )}. \end{aligned}$$

9 $$\begin{aligned} ReZG_{2}(G)= \sum \limits _{\vartheta \varpi \in E(BS_b)}\frac{\Im (\vartheta )\times \Im (\varpi )}{\Im (\vartheta )+\Im (\varpi )}. \end{aligned}$$

10 $$\begin{aligned} ReZG_{3}(G)= \sum \limits _{\vartheta \varpi \in E(BS_b)}\big (\Im (\vartheta ) \times \Im (\varpi ))(\Im (\vartheta )+\Im (\varpi )\big ). \end{aligned}$$

Balaban^29,30^ introduced the Balaban index fora graph *G* with order $\acute{b}$ and size $\acute{l}$ as:

11 $$\begin{aligned} J(G)= \bigg (\frac{\acute{b}}{\acute{b}-\acute{l}+2}\bigg )\left[ \sum \limits _{\vartheta \varpi \in E(BS_b)}\frac{1}{\sqrt{\Im (\vartheta )\times \Im (\varpi )}}\right] . \end{aligned}$$

## Structure of benzyl sulfamoyl network $BS_b$

In the field of materials sciences because of its unique properties, benzyl sulfamoyl $BS_b$ has attained remarkable attentiveness. Dichalcogenides (*TMDCs*), associated with the family of transition metals, are layered materials with a general formula of $MX_b$. $BS_b$ consists of a layered crystal structure containing sulfur atoms that merge between two layers of benzyl sulfamoyl atoms^31^. Various method is used to recognize benzyl sulfamoyl, such as chemical vapor deposition and transport, also hydrothermal synthesis is used for this purpose^32^. Since benzyl sulfamoyl has perceptible magnetic and electrical properties, it has attained abundant interest in the field of materials research. Superconductivity and magnetism of benzyl sulfamoyl are combined displayed, making it a type-II superconductor. Because of this characteristic, it is an advantageous material for several uses, such as energy storage, quantum computing, and spintronics.

**Figure 1:**
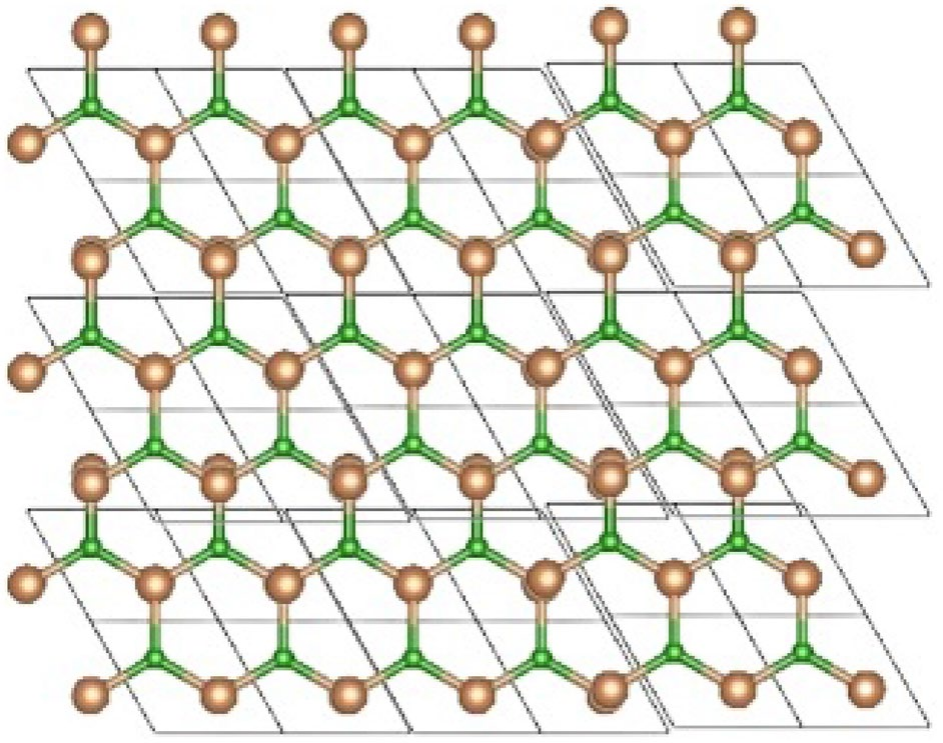
The structure of benzyl sulfamoyl $BS_b$ for $m=n=5$.

The benzyl sulfamoyl $BS_b$ formulas are obtained by initially using the unit cell as shown in Fig. 1. To compute the vertices, we use Matlab software to generalize these formulas for vertices together with Tables 1. The partition of edges for benzyl sulfamoyl $BS_b$ will be taken as follows to determine its topological indices: The partition of edges for benzyl sulfamoyl $BS_b$ with (*m*, *n*) is always greater or equal to 1 that are shown in Table 2. The set of edges is divided into three sets, let’s say $E_1$, $E_2$, and $E_3$, concerning the degree of every vertices and edges. The order and size of $BS_b$ is $8mn+2m+2n$ and 12*mn*, respectively.

**Table 1:** Vertex partition of $BS_b$.

$\Im (\vartheta )$	Frequency	Set of vertices
1	$2m+1+2n$	$V_{1}$
2	$-1+2(m+n)$	$V_{2}$
3	$2mn-2(m+n-1)$	$V_{3}$

**Table 2:** Edge partition of $BS_b$.

$(\Im (\vartheta ),\, \Im (\varpi ))$	Frequency	Set of edges
(1,3)	$2m+1+2n$	$E_{1}$
(2,3)	$4(-1+m+n)$	$E_{2}$
(3,3)	$12mn-6(m+n)+3$	$E_{3}$

In the view of Eq. (1) and Table 2, we prove the following theorems as below:

## Results for benzyl sulfamoyl network $BS_b$

### Theorem 1

*The General Randic indices for benzyl sulfamoyl network* $BS_b$ *are*:

$$\begin{aligned} R_1(BS_b)&= 108mn - 24m -24n+6,\\ R_{-1}(BS_b)&= 1.3333mn+0.6667m+0.6667n,\\ R_{\frac{1}{2}}(BS_b)&= 36mn-4.7379m+4.7379n+0.9341,\\ R_{-\frac{1}{2}}(BS_b)&= 4mn +0.7876m + 0.7876n-0.0556. \end{aligned}$$

### Proof

The derivations for General Randic indices using Table 2 are given as:

$$\begin{aligned} R_\alpha (G)&= \sum \limits _{\vartheta \varpi \in E(BS_b)}(\Im (\vartheta )\times \Im (\varpi ))^\alpha ;\, \, \, \ \alpha = 1,\, -1,\, \frac{1}{2},\, \frac{-1}{2} \\ {\textbf {For}}\,\,\, \alpha&= 1;\\ R_1(BS_b)&= \sum \limits _{i=1}^{3}\sum \limits _{\vartheta \varpi \in E_{i}(BS_b)}(\Im (\vartheta ) \times \Im (\varpi ))^{1}\\&= (1\times 3)(2m+1+2n)+(3\times 2)(4(-1+m+n))+(3\times 3)(12mn-6(m+n)+3)\\&= 108mn - 24m -24n+6. \\ {\textbf {For}}\,\,\, \alpha&= -1;\\ R_{-1}(BS_b)&= \sum \limits _{i=1}^{3}\sum \limits _{\vartheta \varpi \in E_{i}(BS_b)}(\Im (\vartheta )\times \Im (\varpi ))^{-1}\\&= \frac{1}{(1\times 3)}(2m+1+2n)+\frac{1}{(3\times 3)}(4(-1+m+n))+\frac{1}{(3\times 3)}(12mn-6(m+n)+3)\\&= 1.3333mn+0.6667m+0.6667n. \\ {\textbf {For}}\,\,\, \alpha&= \frac{1}{2};\\ R_{\frac{1}{2}}(BS_b)&= \sum \limits _{i=1}^{3}\sum \limits _{\vartheta \varpi \in E_{i}(BS_b)}(\Im (\vartheta )\times \Im (\varpi ))^{\frac{1}{2}}\\ R_{\frac{1}{2}}(BS_b)&= \sqrt{(1\times 3)}(2m+1+2n)+\sqrt{3\times 2}(4(-1+m+n))+\sqrt{(3\times 3)}(12mn-6(m+n)+3)\\&= 36mn-4.7379m+4.7379n+0.9341. \\ {\textbf {For}}\,\,\, \alpha&= -\frac{1}{2};\\ R_{-\frac{1}{2}}(BS_b)&= \sum \limits _{i=1}^{3}\sum \limits _{\vartheta \varpi \in E_{i}(BS_b)}\frac{1}{\sqrt{(\Im (\vartheta )\times \Im (\varpi ))}}\\&= \frac{1}{\sqrt{(3 \times 1)}}(2m+1+2n)+\frac{1}{\sqrt{2 \times 3}}(4(-1+m+n))+\frac{1}{\sqrt{(3 \times 3)}}(12mn-6(m+n)+3)\\&= 4mn +0.7876m + 0.7876n-0.0556. \end{aligned}$$

**Figure 2:**
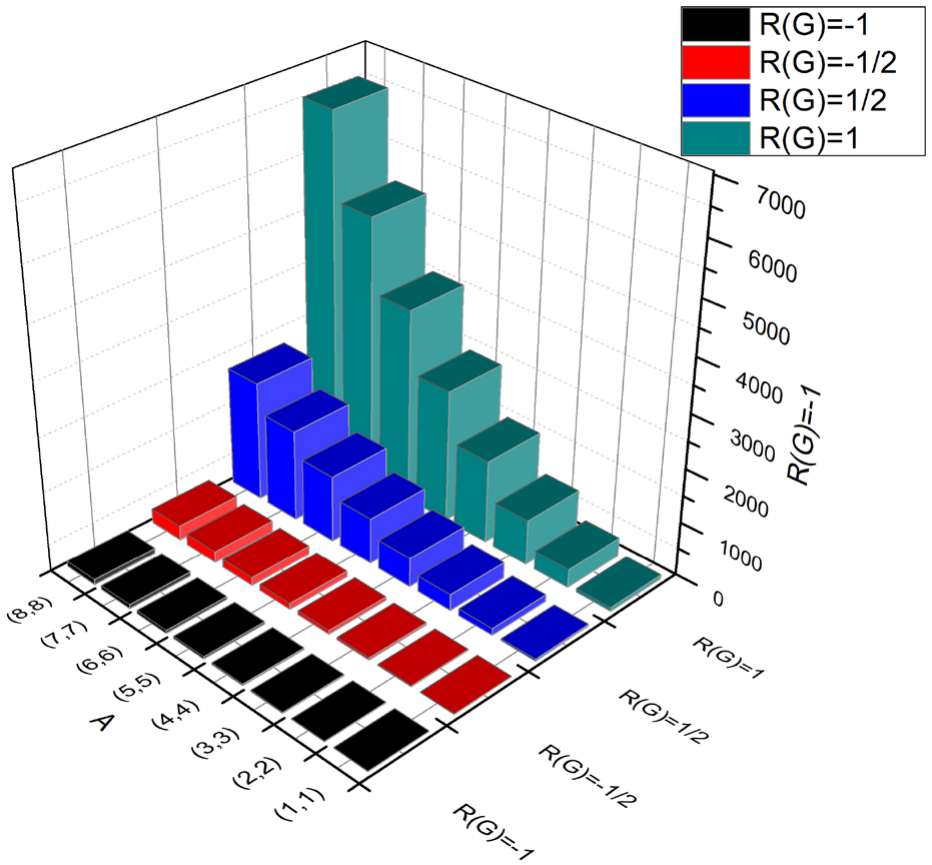
Graphical observation between ${R_{1}(BS_b)}$,${R_{-1}(BS_b)}$,${R_{\frac{1}{2}}(BS_b)}$ and ${R_{\frac{-1}{2}}(BS_b)}$.

To identify the complexity of the topological index of a graph, a mathematical symbol is used, and shown in Fig. 2 is the Randic index. Table 3 shows a numerical observation of the Randic indices as well.

**Table 3:** Numerical observation of $R_1(BS_b)$,  $R_{-1}(BS_b)$,  $R_{\frac{1}{2}}(BS_b)$,  and $R_{\frac{-1}{2}}(BS_b)$.

[*m*, *n*]	$R_{1}(BS_b)$	$R_{-1}(BS_b)$	$R_{\frac{1}{2}}(BS_b)$	$R_{\frac{-1}{2}}(BS_b)$
[1, 1]	66	2.6667	27.4583	5.5196
[2, 2]	342	8	125.9825	19.0948
[3, 3]	834	16	296.5067	40.67
[4, 4]	1542	26.6667	539.0309	70.2452
[5, 5]	2466	40	853.5551	107.8204
[6, 6]	3606	56	1240.0793	153.3956
[7, 7]	4962	74.6667	1698.6035	206.9708
[8, 8]	6534	96	2229.1277	268.546

$$\square$$

### Theorem 2

*The* *ABC* *index*, *GA* *index*, $M_{1}$ *index and* $M_{2}$ *index for benzyl sulfamoyl network* $BS_b$ *are*:

$$\begin{aligned} ABC(BS_b)&= 8mn+0.4614m+0.4614-0.01193,\\ GA(BS_b)&= 12mn-0.3488m-0.3488n-0.05315,\\ M_{1}(BS_b)&= 72mn-8m-8n+2,\\ M_{2}(BS_b)&= 108mn-24m-24n+6. \end{aligned}$$

### Proof

The derivations for topological indices using Table 2 are given as:

The *ABC* index is derived as:

$$\begin{aligned} ABC(BS_b)&= \sum \limits _{i=1}^{3}\sum \limits _{\vartheta \varpi \in E_{i}(BS_b)}\sqrt{\frac{(\Im (\vartheta )+\Im (\varpi )-2)}{\Im (\vartheta ) \times \Im (\varpi )}}\\&= \sqrt{\frac{1+3-2}{(3 \times 1)}}(2m+1+2n)+\sqrt{\frac{2+3-2}{(2 \times 3)}}(4(-1+m+n))+\sqrt{\frac{3+3-2}{(3 \times 3)}}(12mn-6(m+n)+3)\\&= 8mn+0.4614m+0.4614-0.01193. \end{aligned}$$

The *GA* index is derived as:

$$\begin{aligned} GA(BS_b)&= \sum \limits _{i=1}^{3}\sum \limits _{\vartheta \varpi \in E_{i}(BS_b)}\frac{2\sqrt{\Im (\vartheta )\times \Im (\varpi )}}{\Im (\vartheta )+\Im (\varpi )}\\&= \frac{2\sqrt{3 \times 1}}{(3+1)}(2m+1+2n)+\frac{2\sqrt{2 \times 3}}{(3+3)}(4(-1+m+n))+ \frac{2\sqrt{3 \times 3}}{(3+3)}(12mn-6(m+n)+3)\\&= 12mn-0.3488m-0.3488n-0.05315. \end{aligned}$$

The first Zagreb index derived as:

$$\begin{aligned} M_{1}(BS_b)&= \sum \limits _{i=1}^{3}\sum \limits _{\vartheta \varpi \in E_{i}(BS_b)}(\Im (\vartheta )+\Im (\varpi ))\\&= (3+3)(2m+1+2n)+(2+3)(4(-1+m+n))+(3+3)(12mn-6(m+n)+3)\\&= 72mn-8m-8n+2. \end{aligned}$$

The second Zagreb index derived as:

$$\begin{aligned} M_{2}(BS_b)&= \sum \limits _{i=1}^{3}\sum \limits _{\vartheta \varpi \in E_{i}(BS_b)}(\Im (\vartheta )\times \Im (\varpi ))\\&= (3 \times 1)(2m+1+2n)+(2 \times 3)(4(-1+m+n))+(3 \times 3)(12mn-6(m+n)+3)\\&= 108mn-24m-24n+6. \end{aligned}$$

**Figure 3:**
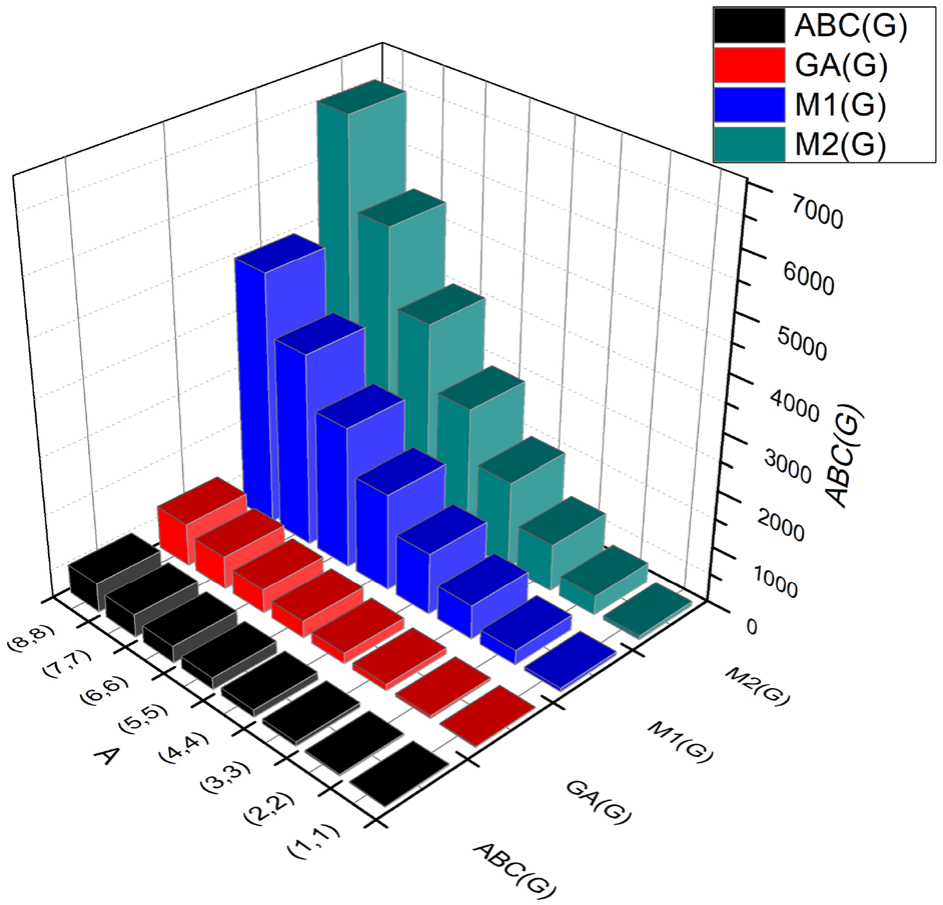
Graphical observation between $ABC(BS_b)$,  $GA(BS_b)$,  $M_{1}(BS_b)$ and $M_{2}(BS_b)$.

The numerical observation and graphical representations of the $ABC(BS_b)$, $GA(BS_b)$, $M_1(BS_b)$, and $M_2(BS_b)$, respectively, are shown in Table 4 and Fig. 3.

**Table 4:** Numerical observation of $ABC(BS_b)$,  $GA(BS_b)$,  $M_{1}(BS_b)$ and $M_{2}(BS_b)$.

[*m*, *n*]	$ABC(BS_b)$	$GA(BS_b)$	$M_1(BS_b)$	$M_2(BS_b)$
[1, 1]	8.91367	11.2494	58	66
[2, 2]	33.8391	46.5521	258	342
[3, 3]	74.7646	105.8547	602	834
[4, 4]	131.6901	189.1573	1090	1542
[5, 5]	204.6156	296.4599	1722	2466
[6, 6]	293.5411	427.7625	2498	3606
[7, 7]	398.4667	583.0651	3418	4962
[8, 8]	519.3921	762.3677	4482	6534

$$\square$$

### Theorem 3

*That* *HM* *index*, *F* *index*, $PM_{1}$ *index and* $PM_{2}$ *index for benzyl sulfamoyl network* $BS_b$ *are*:

$$\begin{aligned} HM(BS_b)&= 432mn-84m-84n+24,\\ F(BS_b)&= 216mn-36m-36n+12. \end{aligned}$$

### Proof

The derivations for topological indices using Table 2 are given as:

The derivations of Hyper Zagreb index:

$$\begin{aligned} HM(BS_b)&= \sum \limits _{i=1}^{3}\sum \limits _{\vartheta \varpi \in E_{i}(BS_b)}(\Im (\vartheta )+\Im (\varpi ))^{2}\\&= (1+3)^{2}(2m+1+2n)+(2+3)^{2}(4(-1+m+n))+(3+3)^{2}(12mn-6(m+n)+3)\\&= 432mn-84m-84n+24. \end{aligned}$$

The Forgotten index is derived as:

$$\begin{aligned} F(BS_b)&= \sum \limits _{i=1}^{3}\sum \limits _{\vartheta \varpi \in E_{i}(BS_b)}(\Im (\vartheta )^{2}+\Im (\varpi )^{2})\\&= (3^{2}+1^{2})(2m+1+2n)+(2^{2}+3^{2})(4(-1+m+n))+(3^{2}+3^{2})(12mn-6(m+n)+3)\\&= 216mn-36m-36n+12. \end{aligned}$$

**Figure 4:**
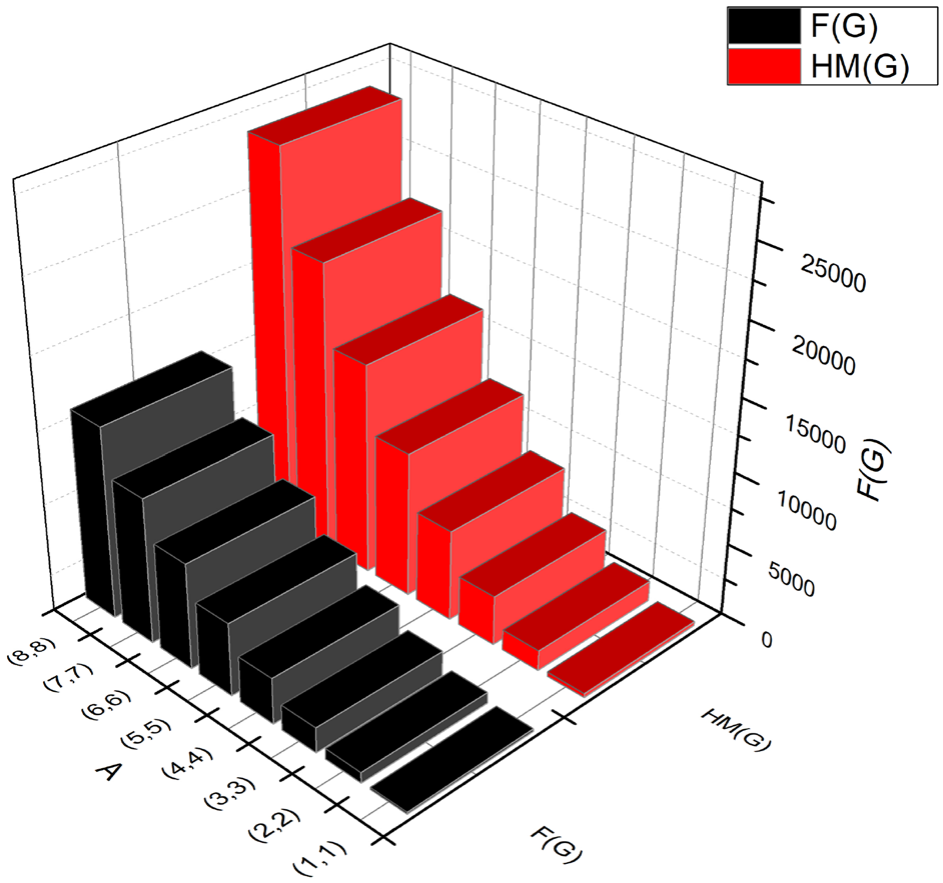
Graphical observation between $HM(BS_b)$, $F(BS_b)$.

The following Table 5 and Fig. 4 represented the graphical and numerical observations of *HM* and *F*, respectively.

**Table 5:** Graphical observation between $HM(BS_b)$, $F(BS_b)$.

[*m*, *n*]	$HM(BS_b)$	$F(BS_b)$
[1, 1]	288	156
[2, 2]	1416	732
[3, 3]	34,082	1740
[4, 4]	6264	3180
[5, 5]	9984	5052
[6, 6]	14,568	7356
[7, 7]	20,016	10,092
[8, 8]	26,328	13,260

$$\square$$

### Theorem 4

*The* *J* *index*, $ReZG_{1}$ *index*, $ReZG_{2}$ *index and* $ReZG_{3}$ *index for benzyl sulfamoyl network* $BS_b$ *are*:

$$\begin{aligned} J(BS_b)&= \left( \frac{12mn}{4mn-2m-2n+2}\right) (4mn+0.7876m+0.7876n-0.0556),\\ ReZG_{1}(BS_b)&= 8mn+2m+2n,\\ ReZG_{2}(BS_b)&= 18mn-2.7m-2.7n+0.45,\\ ReZG_{3}(BS_b)&= 648mn-180m-180n+54. \end{aligned}$$

### Proof

The derivations for topological indices using Table 2 are given as:

The Balaban index is derived as:

$$\begin{aligned} J(BS_b)&= \left( \frac{\acute{b}}{\acute{b}-\acute{l}+2}\right) \bigg [\sum _{i=1}^{3}\sum \limits _{\vartheta \varpi \in E_{i}(BS_b)}\frac{1}{\sqrt{\Im (\vartheta ) \times \Im (\varpi )}}\bigg ]\\&= \left( \frac{12mn}{12mn-8mn-2m-2n+2}\right) \bigg [\frac{1}{\sqrt{(3 \times 1)}} \times (2m+1+2n)\\ &\quad +\frac{1}{\sqrt{(2 \times 3)}} \times (4(-1+m+n))+\frac{1}{\sqrt{(3 \times 3)}} \times (12mn-6(m+n)+3)\bigg ]\\&= \left( \frac{12mn}{4mn-2m-2n+2}\right) (4mn+0.7876m+0.7876n-0.0556). \end{aligned}$$

We formulate the First, Second and Third Redefined Zagreb index as follows:

$$\begin{aligned} ReZG_{1} (BS_b)&= \sum \limits _{i=1}^{3}\sum \limits _{\vartheta \varpi \in E_{i}(BS_b)}\frac{\Im (\vartheta )+\Im (\varpi )}{\Im (\vartheta )\times \Im (\varpi )}\\&= \left( \frac{3+1}{3 \times 1}\right) (2m+1+2n)+\left( \frac{2+3}{2 \times 3}\right) (4(-1+m+n))\\ &\quad + \left( \frac{3+3}{3 \times 3}\right) (12mn-6(m+n)+3)\\& = 8mn+2m+2n. \\ ReZG_{2}(BS_b)&= \sum \limits _{i=1}^{3}\sum \limits _{\vartheta \varpi \in E_{i}(BS_b)}\frac{\Im (\vartheta )\times \Im (\varpi )}{\Im (\vartheta )+\Im (\varpi )}\\&= \left( \frac{3 \times 1}{3+1}\right) (2m+1+2n)+\left( \frac{2 \times 3}{2+3}\right) (4(-1+m+n))\\{} & \quad +\left( \frac{3 \times 3}{3+3}\right) (12mn-6(m+n)+3)\\&= {} 18mn-2.7m-2.7n+0.45. \\ ReZG_{3}(BS_b)&= {} \sum \limits _{i=1}^{3}\sum \limits _{\vartheta \varpi \in E_{i}(BS_b)}\big (\Im (\vartheta )\times \Im (\varpi )\big )\big ( \Im (\vartheta )+\Im (\varpi )\big ) \\&= {} (1\times 3)\times (3+1)(2m+1+2n)+(3\times 2)\times (2+3)(4(-1+m+n))\\{} & \quad \times (3\times 2) \times (3+3)(12mn-6(m+n)+3)\\&= {} 648mn-180m-180n+54. \end{aligned}$$

**Figure 5:**
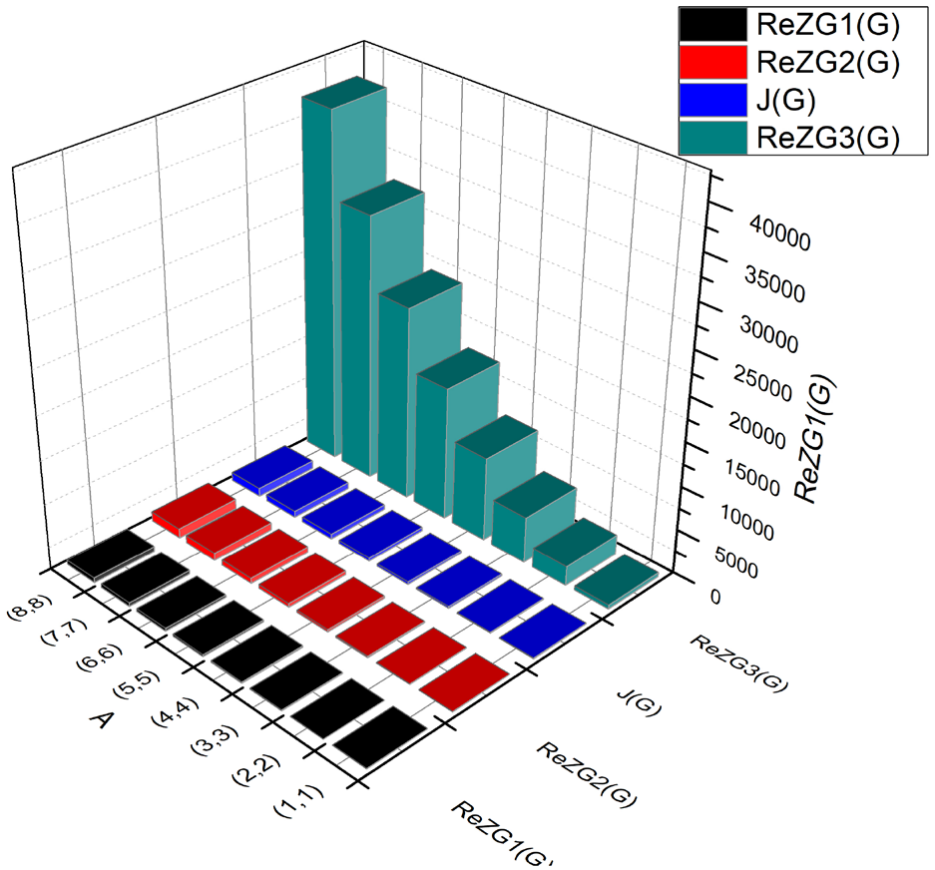
Graphical observation of $J(BS_b)$,$ReZG_{1}(BS_b)$, $ReZG_{2}(BS_b)$ and $ReZG_{3}(BS_b)$.

The $J(BS_b)$,$ReZG_{1}(BS_b)$, $ReZG_{2}(BS_b)$ and $ReZG_{3}(BS_b)$ are represented graphically and numerically in Table 6 and Fig. 5.

**Table 6:** Numerical Pattern of $J(BS_b)$,$ReZG_{1}(BS_b)$, $ReZG_{2}(BS_b)$ and $ReZG_{3}(BS_b)$.

[*m*, *n*]	$J(BS_b)$	$ReZG_1(BS_b)$	$ReZG_2(BS_b)$	$ReZG_3(BS_b)$
[1, 1]	33.1176	12	13.05	486
[2, 2]	91.65504	40	61.65	2214
[3, 3]	168.93692	84	146.25	5238
[4, 4]	269.7416	144	266.85	9558
[5, 5]	394.4649	220	423.45	15,174
[6, 6]	543.171305	312	616.05	22,086
[7, 7]	715.8755	420	844.65	30,294
[8, 8]	912.5811	544	1109.25	39,798

$$\square$$

## Analysis of formation of heat via Rational curve fitting

A mathematical model’s parameters are found using a statistical technique called curve fitting, which enables the model to approximately describe the observed data. This part of paper consist the idea of the illumination of entropy and formation of heat(Enthalpy) *HOF* of benzyl sulfamoyl $BS_b$. The standard molar enthalpy of benzyl sulfamoyl $BS_b$ at temperature 298.15*K* see details^33^. To calculate formation of heat (HOF) for unlike formula units Mathematical formula is given as:

12 $$\begin{aligned} HOF = \frac{Standard \,\ Molar\,\ HOF}{Avogadro\text{'}s \,\ Number} \times \,\ Formula \,\ Units \end{aligned}$$

Where, $Avogadro's \,\ Number = 6.02214076 \times 10^{23}\, \text {mol}^{-1}$^19^. One mole of any substance is equal to $6.02214076 \times 10^{23} \, \text {mol}^{-1}$ of that material (such as atoms, molecules, or ions). Each material is in its normal state when a mole of a chemical is created by combining its constituent elements, and the amount of energy absorbed or released is measured by the formation of heat (*HOF*). This is calculated in kilojoules per mole, or KJ/mol. It can also be described as having a normal formation of heat or a specific heat capacity^34^.

**Figure 6:**
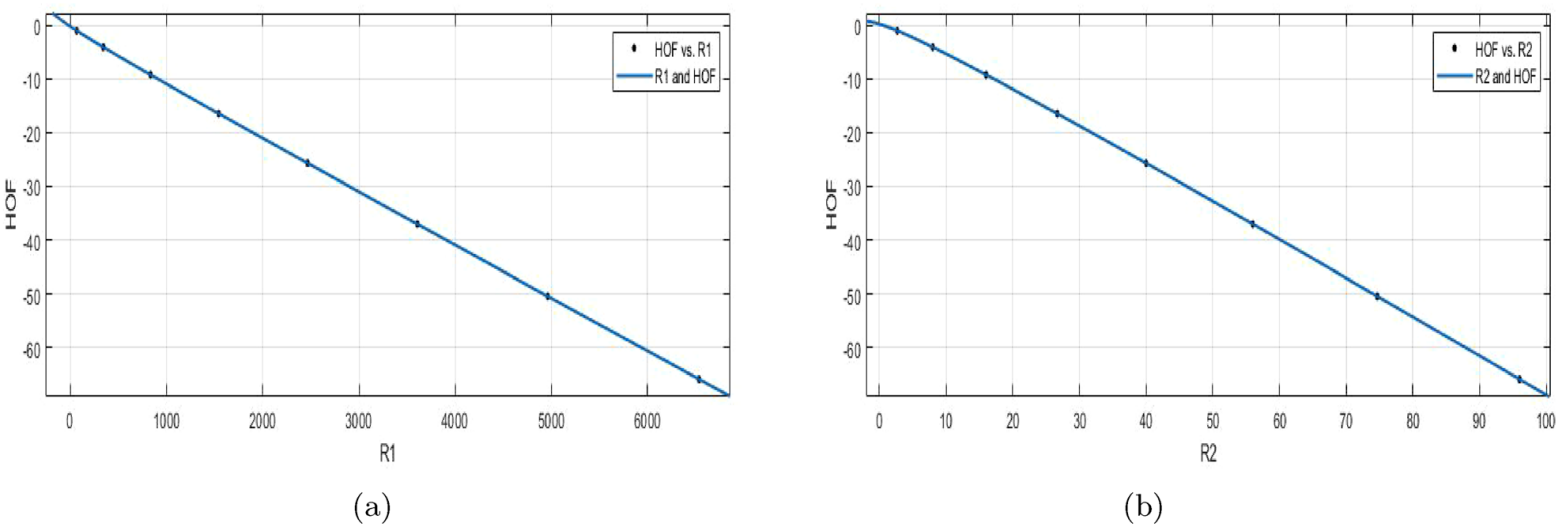
(**a**) ${\mathfrak {R}}_{{c}{f}}$ between HOF of $R_{1}(BS_b)$; (**a**) ${\mathfrak {R}}_{{c}{f}}$ between HOF of $R_{-1}(BS_b)$.

**Figure 7:**
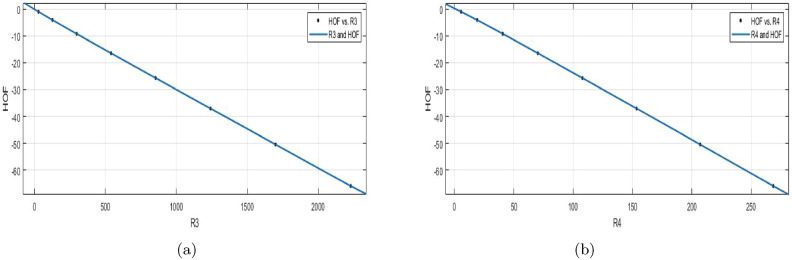
(**a**) ${\mathfrak {R}}_{{c}{f}}$ between HOF of $R_{\frac{-1}{2}}(BS_b)$; (**b**) ${\mathfrak {R}}_{{c}{f}}$ between HOF of $R_{\frac{-1}{2}}(BS_b)$.

**Figure 8:**
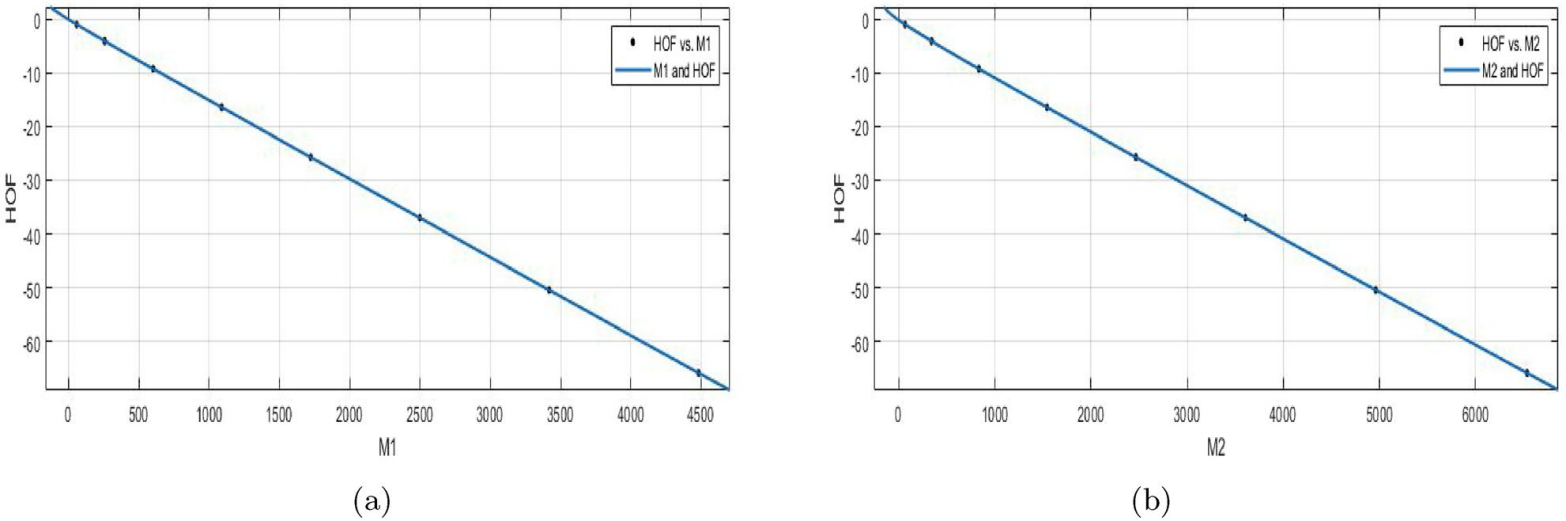
(**a**) ${\mathfrak {R}}_{c}{f}$ between *HOF* of $M_{1}(BS_b)$; (**b**) ${\mathfrak {R}}_{c}{f}$ between *HOF* of $M_{2}(BS_b)$.

**Figure 9:**
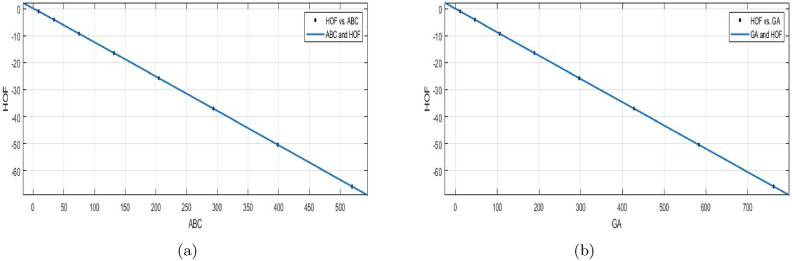
(**a**) ${\mathfrak {R}}_{c}{f}$ between HOF of ${ABC}(BS_b)$; (**b**) ${\mathfrak {R}}_{c}{f}$ between HOF of $GA(BS_b)$.

**Figure 10:**
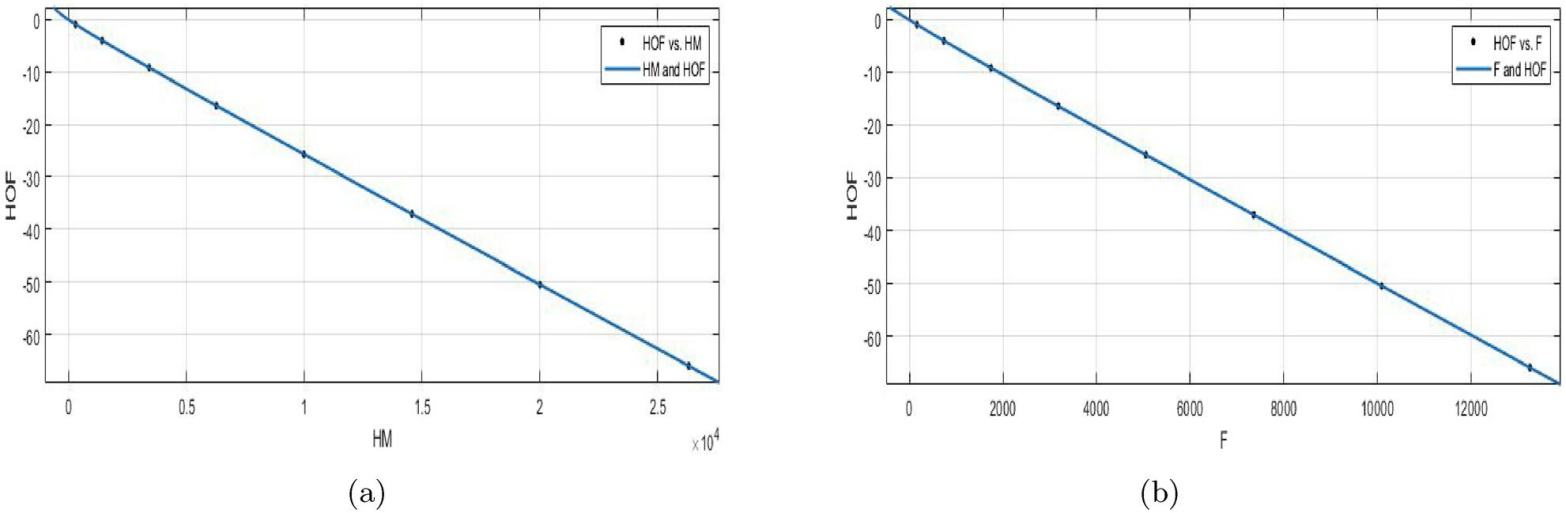
(**a**) ${\mathfrak {R}}_{c}{f}$ between *HOF* of ${HM}(BS_b)$; (**b**) ${\mathfrak {R}}_{c}{f}$ between *HOF* of ${F}(BS_b)$.

**Figure 11:**
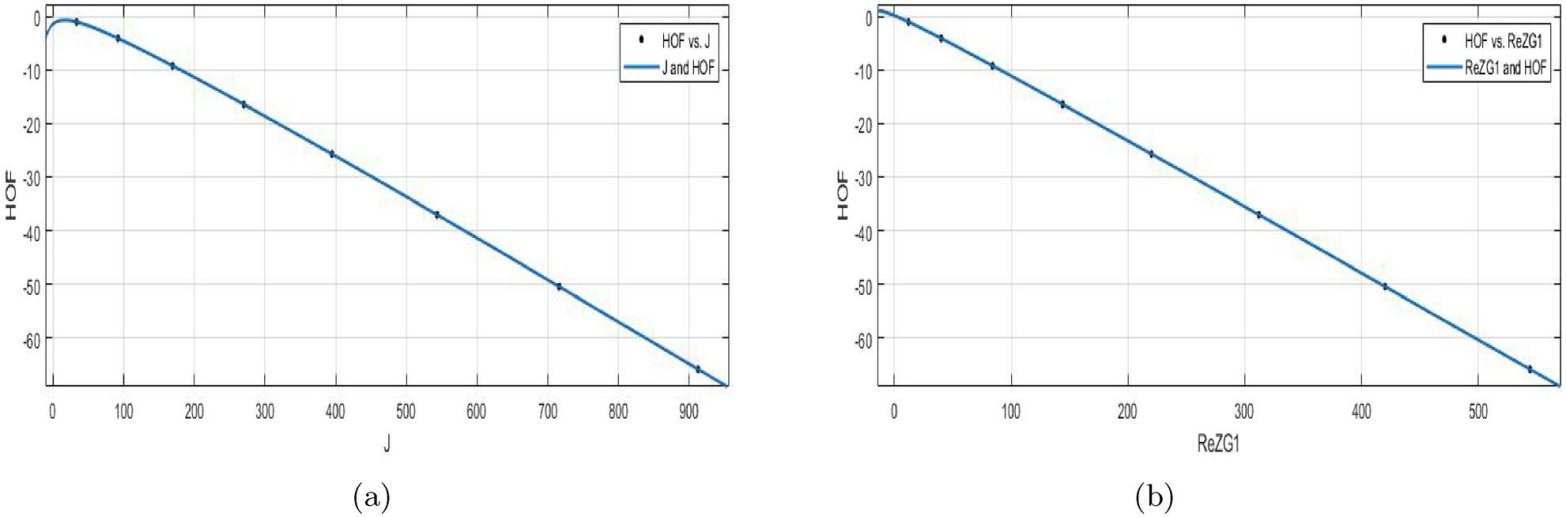
(**a**) ${\mathfrak {R}}_{c}{f}$ between *HOF* of ${J}(BS_b)$; (**b**) ${\mathfrak {R}}_{c}{f}$ between *HOF* of $ReZG_1(BS_b)$.

**Figure 12:**
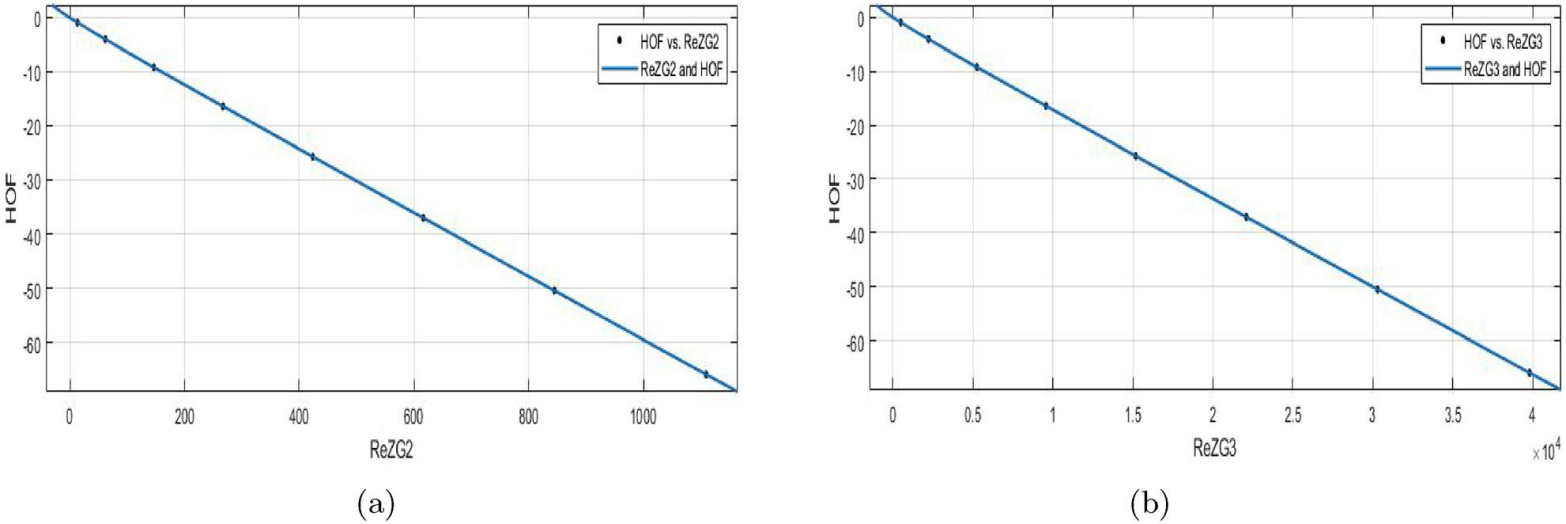
(**a**) ${\mathfrak {R}}_{c}{f}$ between *HOF* of $ReZG_2(BS_b)$; (**b**) ${\mathfrak {R}}_{c}{f}$ between *HOF* of $ReZG_3(BS_b)$.

MATLAB’s Power built-in function is used to create models between formation of heat and each particular entropy since it provides a lower *RMSE* value for the best fit. The mean squared error (*RMSE*), the sum of squared errors (*SSE*), and $R^2$ are the accuracy metrics that are employed. First, calculate the values of various indices using the Shanon entropy formula. Then we use the Maple software to determine the entropy’s graphical behavior and computed numerical values. Furthermore, curves were fitted between the formation of heat values and a number of related entropies. The models for the relationship between indices vs. *HOF* that were described are shown below. Graphical behaviour is presented in Figs. 6, 7, 8, 9, 10, 11, and 12.

- **HOF using** $$R_1(BS_b)$$

$$\begin{aligned} f(R_1) = \frac{( p1\times R_1^2 + p2\times R_1 + p3 )}{ (R_1^2+q1\times R_1+q2)} \end{aligned}$$

$R_1$ is standardized (normalized) by mean value of 2544 and the standard deviation is 2324. The measures are: $p1 = -6194(-8791,-3598)$, $p2= -1.538e{+}04(-2.234e{+}04,-8411)$, $p3 = -9424(-1.395e{+}04,-4893)$, $q1 = 269.3(155.4,383.3)$, $q2 =355.6(184.6,526.5)$. In all curve fittings, the confidence bound is $95\%$.

$$SSE{:}\, 0.0001569$$, *R*-$$square{:}\, 1$$, $$Adjusted{:}\, 1$$, and $$RMSE{:}\, 0.007233$$

- **HOF using** $$R_{-1}(BS_b)$$

$$\begin{aligned} f(R_{-1}) = \frac{p1\times R_{-1}^2+p2\times R_{-1}+p3}{R_{-1}^4+q1\times R_{-1}^3+q2\times R_{-1}^2+q3\times R_{-1}+q4} \end{aligned}$$

Where $R_{-1}$ is NBM by $\mu =40$ and standard deviation is 33.31. The measures are: $p1 =-3.629e{+}05(-1.159e{+}08, 1.152e{+}08)$, $p2 = -9.148e{+}05 (-2.919e{+}08, 2.9e{+}08)$, $p3 = -5.745e{+}05 (-1.831e{+}08, 1.819e{+}08)$, $q1 = 37.14 (-1.264e{+}04, 1.271e{+}04)$, $q2 = -199.8 (-6.453e{+}04, 6.413e{+}04)$, $q3 = 1.529e{+}04 (-4.853e{+}06, 4.883e{+}06)$, $q4 =2.233e{+}04 (-7.071e{+}06, 7.116e{+}06)$.

$$SSE{:}\, 5.642e{-}05$$, *R*-$$square{:}\, 1$$, *Adjusted* *R*-$$square{:}\, 1$$, and $$RMSE{:}\, 0.007511$$.

- **HOF using** $${R_{\frac{1}{2}}(BS_b)}$$

$$\begin{aligned} f({R_\frac{1}{2})} = \frac{(\ p1\times R_\frac{1}{2} + p2)}{R_\frac{1}{2}^5+q1\times R_\frac{1}{2}^4 + q2\times R_\frac{1}{2}^3+q3\times R_\frac{1}{2}^2 +q4\times R_\frac{1}{2}+q5)} \end{aligned}$$

In the above formula $R_{-1}$ is NBM 40 and standard deviation 33.31. The measures are: $p1 =-3.629e{+}05\,(-1.159e{+}08, 1.152e{+}08)$, $p2 = -9.148e{+}05 (-2.919e{+}08, 2.9e{+}08)$, $p3 = -5.745e{+}05 (-1.831e{+}08, 1.819e{+}08)$, $q1 = 37.14 (-1.264e{+}04, 1.271e{+}04)$, $q2 = -199.8 (-6.453e{+}04, 6.413e{+}04)$, $q3 = 1.529e{+}04 (-4.853e{+}06, 4.883e{+}06)$, $q4 =2.233e{+}04 (-7.071e{+}06, 7.116e{+}06)$.

$$SSE{:}\, 0.0001191$$, *R*-$$square{:}\, 1$$, *Adjusted* *R*-$$square{:}\, 1$$, and $$RMSE{:}\, 0.01091$$.

- **HOF using** $$R_{\frac{-1}{2}}(BS_b)$$

$$\begin{aligned} f({R_\frac{-1}{2})} = \frac{(p1\times R_\frac{-1}{2} + p2)}{(R_\frac{-1}{2}^4 + q1 \times R_\frac{-1}{2}^3+ q2 \times R_\frac{-1}{2}^2 +q3\times R_\frac{-1}{2} +q4)} \end{aligned}$$

In which $R_\frac{-1}{2}$ is NBM 109 and standard deviation 94.1. The measures are: $p1 = -5466 (-1.534e{+}04, 4409)$, $p2 = -6277 (-1.76e{+}04, 5045)$, $q1 = -3.389 (-5.493, -1.286)$, $q2 = 4.521(-2.579, 11.62)$, $q3= -5.784(-17.26, 5.692)$, $q4 = 241(-194, 676)$. $SSE{:}\, 0.000811$, *R*-$square{:}\, 1$, *Adjusted* *R*-$square{:}\, 1$, and $RMSE{:}\, 0.02014$.

It is important to note that the relationship between the Randic index and heat of formation is likely complex and may depend on other factors besides branching. The Randi$\acute{c}$ index for a specific molecule corresponds to its value, and the coefficients control how the Randi$\acute{c}$ index are related with the formation of heat. In the graph Fig. 7, the x-axis represents the Randic index (R1) and the y-axis represents the heat of formation (HOF). The data points show a positive correlation between the two variables. This means that as the Randic index increases (indicating more branching), the heat of formation becomes more positive (indicating a more stable molecule).

- **HOF using** $$M_{1} (BS_b)$$

$$\begin{aligned} f(M_1) = \frac{( p1\times M_1^4 + p2\times M_1^3 + p3\times M_1^2 + p4\times M_1+p5)}{(M_1 + q1)} \end{aligned}$$

In which $M_1$ is NBM 1766 and standard deviation 1588. The measures are: $p1 = -0.01189 (-0.02355, -0.0002294)$, $p2 = 0.05759 (0.04042, 0.07477)$,

$$p3 = -23.06 (-23.08, -23.05)$$, $$p4 = -56.41 (-58.1, -54.71)$$, $$p5 = -34.13 (-36.06, -32.2)$$, $$q1 = 1.294 (1.221, 1.367)$$.

$$SSE{:}\, 3.005e{-}06$$, *R*-$$square{:}\, 1$$, *Adjusted* *R*-$$square{:}\, 1$$, and $$RMSE{:}\, 0.001226$$.

- **HOF using** $$M_{2}(BS_b)$$

$$\begin{aligned} f(M_2) = \frac{(p1\times M_2^5 + p2\times M_2^4 + p3\times M_2^3 + p4\times M_2^2+p5\times M_2 +p6)}{(M_2 + q1)} \end{aligned}$$

In which $M_2$ is NBM 2544 and standard deviation 2324. The measures: $p1 =0.01287$ (− 0.06615, 0.09189), $p2 = -~0.04554 (-~0.1632, 0.07208)$, $p3 = 0.1065 (-~0.008764, 0.2217)$, $p4 = -22.88 (-22.94, -22.81)$, $p5 = -54.91 (-60.15, -49.68)$, $p6 = -32.5 (-38.45, -26.55)$, $q1= 1.226 (1.001, 1.451)$.

$$SSE{:}\, 2.979e{-}06$$, *R*-$$square{:}\, 1$$, *Adjusted* *R*-$$square{:}\, 1$$, and $$RMSE{:}\, 0.001726$$.

The main purpose is to create a mathematical formula or model that can precisely predict the formation of heat of the Zagreb index.

- **HOF using** $$ABC(BS_b)$$

$$\begin{aligned} f(ABC) = \frac{(p1\times ABC^2 + p2\times ABC + p3}{(ABC^2 + q1\times ABC + q2 )} \end{aligned}$$

In which *ABC* is NBM 208.2 and standard deviation 182.9. The measures:

$$p1 = -7.21e{+}04$$ $$(-7.081e{+}05, 5.639e{+}05)$$, $$p2 = -2.099e{+}05$$ $$(-1.996e{+}06, 1.576e{+}06)$$,

$$p3 =-1.454e{+}05$$ $$(-1.353e{+}06, 1.063e{+}06)$$, $$q1 = 3082 (-2.413e{+}04, 3.029e{+}04)$$, $$q2 = 5553$$ $$(-4.059e{+}04, 5.17e{+}04)$$.

$$SSE{:}\, 0.0001287$$, *R*-$$square{:}\, 1$$, *Adjusted* *R*-$$square{:}\, 1$$, and $$RMSE{:}\, 0.00655$$.

The connection between the formation of heat and atom bond connectivity index is examine by using a curve fitting model.

- **HOF using** $$GA(BS_b)$$

$$\begin{aligned} f(GA) = \frac{( p1 \times GA +p2)}{(GA^4+q1\times GA^3 +q2 \times GA^2 + q3\times GA+ q4)} \end{aligned}$$

In which *GA* is NBM 302.8 and standard deviation 269.3. The measures: $p1 = -8.657e{+}05 (-8.952e{+}07, 8.778e{+}07)$, $p2 = -9.75e{+}05 (-1.008e{+}08, 9.887e{+}07)$, $q1 = 29.16 (-3198, 3256)$, $q2 = -105.6 (-1.1e{+}04, 1.079e{+}04)$, $q3 = 162.7 (-1.644e{+}04, 1.676e{+}04)$, $q4 = 3.71e{+}04 (-3.762e{+}06, 3.837e{+}06)$.

$$SSE{:}\, 0.0001158$$, *R*-$$square{:}\, 1$$, *Adjusted* *R*-$$square{:}\, 1$$, and $$RMSE{:}\, 0.007609$$.

If the parameters of the curve fitting model have been established, the model that can be used to predict the formation of heat for geometric arithmetic indicators values inside the range of the data used for curve fitting.

- **HOF using** $$HM(BS_b)$$

$$\begin{aligned} f{(HM)} = \frac{p1\times HM^5 + p2\times HM^4 + p3\times HM^3+p4\times HM^2+p5\times HM+p6)}{(HM +q1)} \end{aligned}$$

In which *HM* is NBM $1.028e{+}04$ and standard deviation 9355. The measures are:

$$p1 =0.01079 (-0.06226, 0.08383)$$, $$p2 =-0.03862 (-0.148, 0.07075)$$,

$$p3 = 0.09268 (-0.01252, 0.1979)$$, $$p4 = -22.92 (-22.98, -22.86)$$,

*p*5 =$$-55.08 (-60.88, -49.28)$$,*p*6 = $$-32.68 (-39.26, -26.09)$$, *q*1= 1.234(0.9853, 1.483).

$$SSE{:}\, 2.473e{-}06$$, *R*-$$square{:}\, 1$$, *Adjusted* *R*-$$square{:}\, 1$$, and $$RMSE{:}\, 0.001573$$.

- **HOF using** $$F(BS_b)$$

$$\begin{aligned} f{(F)} = \frac{(p1\times F^2 + p2\times F + p3)}{(F^2 +q1\times F+ q2)} \end{aligned}$$

In which *F* is NBM 5196 and standard deviation 4706. The measures are:

*p*1 = $$2.861e{+}05 (-2.254e{+}07, 2.311e{+}07)$$, *p*2 = $$7.838e{+}05 (-6.159e{+}07, 6.316e{+}07)$$, *p*3 = $$5.178e{+}05 (-4.061e{+}07, 4.165e{+}07)$$, *q*1 = $$-1.25e{+}04 (-1.01e{+}06, 9.846e{+}05)$$, *q*2 = $$-1.958e{+}04 (-1.575e{+}06, 1.536e{+}06)$$.

$$SSE{:}\, 0.001106$$, *R*-$$square{:}\, 1$$, *Adjusted* *R*-$$square{:}\, 1$$, and $$RMSE{:}\, 0.0192$$.

A relationship between the forgetting index and heat of creation is establish by A curve fitting model . This exploration improves knowledge about the molecule’s energy properties.

- **HOF using** $$J(BS_b)$$

$$\begin{aligned} f{(J)} = \frac{(p1\times J^5 + p2\times J^4 + p3\times J^3 + p4\times J^2+p5\times J+p6)}{(J + q1)} \end{aligned}$$

In which *J* is NBM 391.2 and standard deviation is 312.2. The measures are:

$$p1 = -0.0292 (-0.05179, -0.006602)$$, $$p2=0.1139 (0.08058, 0.1473)$$, $$p3= -0.3123 (-0.3421, -0.2825)$$,

$$p4=-24.4 (-24.42, -24.39)$$,$$p5=-56.85 (-57.66, -56.05)$$, $$p6 = -34.03 (-34.91, -33.14)$$,$$q1= 1.335 (1.301, 1.37)$$.

$$SSE{:}\, 1.864e{-}07$$, *R*-$$square{:}\, 1$$, *Adjusted* *R*-$$square{:}\, 1$$, and $$RMSE{:}\, 0.0004318$$.

The shape of a molecule and topological complexity is measured by the Balaban index, a molecular graph descriptor. The energy change that takes place in the process of the production of a compound and the formation of heat is also measured.

- **HOF using** $$ReZG_{1}(BS_b)$$

$$\begin{aligned} f(ReZG_1) =\frac{(p1\times ReZG_1^5 + p2\times ReZG_1^4 + p3\times ReZG_1^3 +p4\times ReZG_1^2+p5\times ReZG_1+ p6)}{(ReZG_1 + q1)} \end{aligned}$$

In which $ReZG_1$ is NBM 222 and standard deviation 190.2. The measures are:

$$p1= -0.01176 (-0.02673, 0.003214)$$, $$p2=0.04379 (0.01992, 0.06767)$$, $$p3= -0.1095 (-0.1278, -0.09118)$$,

$$p4=-23.66 (-23.66, -23.65)$$,$$p5= -57.08 (-58.82, -55.34)$$, $$p6=-34.66 (-36.6, -32.71)$$, $$q1= 1.334 (1.259, 1.409)$$.

$$SSE{:}\, 7.64e{-}08$$, *R*-$$square{:}\, 1$$, *Adjusted* *R*-$$square{:}\, 1$$, and $$RMSE{:}\, 0.0002764$$.

- **HOF using** $$ReZG_2(BS_b)$$

$$\begin{aligned} f(ReZG_2) =\frac{(p1\times ReZG_2^4 +p2\times ReZG_2^3+p3\times ReZG_2^2+p4\times ReZG_2+p5 )}{(ReZG_2 + q1)} \end{aligned}$$

In which $ReZG_2$ is NBM 435.1 and standard deviation 393.6. The measures are:

$$p1= -0.01704 (-0.03268, -0.0014),$$ $$p2=0.07942 (0.05661, 0.1022),$$

$$p3= -23 (-23.02, -22.98),$$ $$p4= -56.14 (-57.74, -54.54),$$ $$p5=-33.85 (-35.68, -32.02),$$ $$q1=1.281 (1.212, 1.351)$$. $$SSE{:}\, 5.646e{-}06$$, *R*-$$square{:}\, 1$$, *Adjusted* *R*-$$square{:}\, 1$$, and $$RMSE{:}\, 0.00168$$.

- **HOF using** $$ReZG_3(BS_b)$$

$$\begin{aligned} f(ReZG_3) =\frac{(p1 \times ReZG_3^4 + p2 \times ReZG_3^3 + p3 \times ReZG_3^2 + p4 \times ReZG_3+p5 )}{(ReZG_3 + q1)} \end{aligned}$$

In which $ReZG_3$ is NBM $1.561e{+}04$ and standard deviation $1.412e{+}04$. The measures are:

$$p1= -0.01933 (-0.03683, -0.001827),$$ $$p2= 0.08897 (0.06356, 0.1144),$$

$$p3= -22.97 (-22.99, -22.95)$$, $$p4= -56.03 (-57.61, -54.46)$$,

$$p5=-33.74 (-35.54, -31.94)$$, $$q1=1.276(1.208, 1.344)$$.

$$SSE{:}\, 7.195e{-}06$$, *R*-$$square{:}\, 1$$, *Adjusted* *R*-$$square{:}\, 1$$, and $$RMSE{:}\, 0.001897$$.

In the curve fitting process the following steps are used like selecting an suitable model, calculating its parameters using the available data, and evaluate the model’s goodness of fit. so, the fitted curve may be used to forecast the link between the improved Redefined Zagreb index and formation of heat and can also be used for analysis.

## Conclusion

This study presents a comprehensive analysis of the relationship between topological indices and the heat of formation in the benzyl sulfamoyl network using a curve fitting model. We utilized degree counting method, the methods for vertex partition , methods for partition of edges , graph theoretical tools, methods for sum-up the degrees of neighbors , analytic approaches, and combinatorial computing method are the methods to compute our results for $BS_b$. Furthermore, graphical representation of these mathematical results by using Maple we plot and also do mathematical computations using MATLAB. We examine topological indices provide thorough comprehension of the network’s connectivity patterns, characterizing by its distinct characteristics and possible uses . The statistical analysis that are carried out provide a quantitative viewpoint on the observed properties and lay the groundwork for further research, which adds to the larger scientific conversation.

Our results show strong relationships between the chosen topological indices and the formation heat, highlighting the usefulness of these indices in precise thermodynamic feature prediction. Researchers in the domains of materials science and pharmaceuticals can benefit greatly from the created curve fitting models, which provide a strong foundation for calculating the heat of formation based on molecular topology. This article’s results not only highlight the importance of topological indices in materials science investigations but also improve our understanding of the benzyl sulfamoyl network but also. These methods and observation that are represented here open up new directions for advancements in the field as we continue to reveal the molecular details of different materials.

## Data Availability

The datasets used and/or analysed during the current study available from the corresponding author on reasonable request.
